# Intramuscular Aseptic Cyst Syndrome: A Case Report and a Review of the Literature

**DOI:** 10.7759/cureus.87939

**Published:** 2025-07-14

**Authors:** Ioannis Stavrakakis, Emmanouil Kroustalakis, Nikolaos Ritzakis, Chrysostomos Tsatsoulas, George Papazoglou

**Affiliations:** 1 Orthopaedics and Traumatology, Venizeleio and Pananio General Hospital, Heraklion, GRC; 2 Internal Medicine, Venizeleio and Pananio General Hospital, Heraklion, GRC

**Keywords:** aseptic abscesses, aseptic cyst syndrome, intramuscular aseptic abscess, intramuscular cyst, noninfectious cyst

## Abstract

Aseptic cyst syndrome (ACS) is a rare condition frequently associated with a systemic inflammatory response. The clinical presentation closely mimics an infection, making diagnosis and treatment challenging. The most common location of aseptic abscesses is intra-abdominal. Intramuscular involvement is highly uncommon, typically affecting muscles near the trunk. This report presents a case of a male patient who developed multiple aseptic abscesses in the hamstrings and proximal gastrocnemius (GM) muscles. The patient initially underwent surgical debridement and cyst resection, followed by intravenous antibiotic therapy. Although there was a brief period of improvement, a relapse occurred, marked by renewed pain, fever, elevated infection biomarkers, and knee joint effusion. The patient was ultimately treated with corticosteroids, followed by immunosuppressant therapy, resulting in rapid disease improvement. No recurrence was observed at the final follow-up, nine months after symptom onset. A narrative review of the literature on this topic is also included. This case underscores the importance of high clinical suspicion and a multidisciplinary approach in the diagnosis and management of ACS.

## Introduction

Aseptic abscess (AA) formation is rare, and intramuscular localization is highly uncommon. Orphanet (link: https://www.orpha.net/en/disease/detail/54251) reports an incidence of fewer than one in 1000000 people, with approximately 150 cases described in the literature to date. It is frequently associated with inflammatory bowel disease (IBD), rheumatoid arthritis, and other autoimmune diseases [1,2]. Hematologic malignancies such as myelodysplastic syndrome or leukemia may also be linked to the development of AA [3]. The pathophysiology of the condition remains largely unclear. Vessels filled with polymorphonuclear neutrophils (PNNs) are sometimes observed at the periphery of AA lesions, but this may simply reflect the influx of PNNs into the inflammatory site before migrating toward the abscess. The strong association of acute aseptic abscess syndrome (ACS) with autoimmune diseases, along with the predominant neutrophilic component, suggests a key role of neutrophilic innate immunity in this pathological entity [4].

Aseptic abscess syndrome (ACS) is a rare inflammatory disorder characterized by sterile collections of neutrophils that clinically resemble infectious abscesses. Patients often present with fever, localized pain, and elevated inflammatory markers, frequently leading to misdiagnosis and the initiation of antibiotic therapy. However, cultures remain negative, and symptoms typically fail to resolve without immunosuppressive treatment [1,2,5]. The most common location of AA is intra-abdominal, accounting for 65% of cases, with a predominance in the liver and spleen. Skin manifestations occur in 30% of cases [1]. Intramuscular involvement is rare and is even more uncommon in muscles distal to the trunk [1,2,6].

Diagnosis is made after excluding all infectious causes. Cultures and blood investigations are typically negative for bacteria, and antibiotics are ineffective in relieving symptoms [7].

This article presents a unique case of multiple AA in the distal hamstrings and proximal gastrocnemius (GM) muscles of the left leg, an extremely rare location. The patient was successfully treated through the collaboration of orthopedic surgeons, radiologists, internal medicine physicians, gastroenterologists, and rheumatologists.

## Case presentation

A 38-year-old male patient working as a shepherd presented to the emergency department of our institution with pain and swelling in the posterior distal thigh and proximal tibia of the left leg, which had started 16 hours earlier. The patient also reported a fever of up to 39°C. The pain did not subside at rest. No recent injury or wound around the left knee was reported. The patient had undergone a left knee aspiration for joint effusion one month prior to presentation. His past medical history was unremarkable. On examination, a palpable and tender multilobulated mass was identified on the posteromedial aspect of the distal thigh and proximal GM. The range of motion (ROM) of the left knee was normal, and no intra-articular effusion was evident.

The white blood cell (WBC) count was 13200 with 72.8% neutrophils and 16.6% lymphocytes. Hemoglobin (HGB) was 12.3 g/dL. C-reactive protein (CRP) was 15.9 mg/dL (normal <0.5 mg/dL), and erythrocyte sedimentation rate (ESR) was 86 mm/h (Table 1).

**Table 1:** Patient's laboratory test. HGB, hemoglobin; WBC, white blood cells; CRP, C-reactive protein; ESR, erythrocyte sedimentation rate

Parameter	HGB (g/dL)	WBC/μL (neutrophil rate)	CRP (mg/dL)	ESR (mm/1st hour)
Patient’s values on admission (day 0)	12.3	13200 (72.8%)	15.9	86
Patient’s values on discharge (day 21)	13.8	7300 (65%)	1.5	48
Normal values	13.4-17.4	3800-10500 (40-75%)	<0.5	0-20

**Figure 1:**
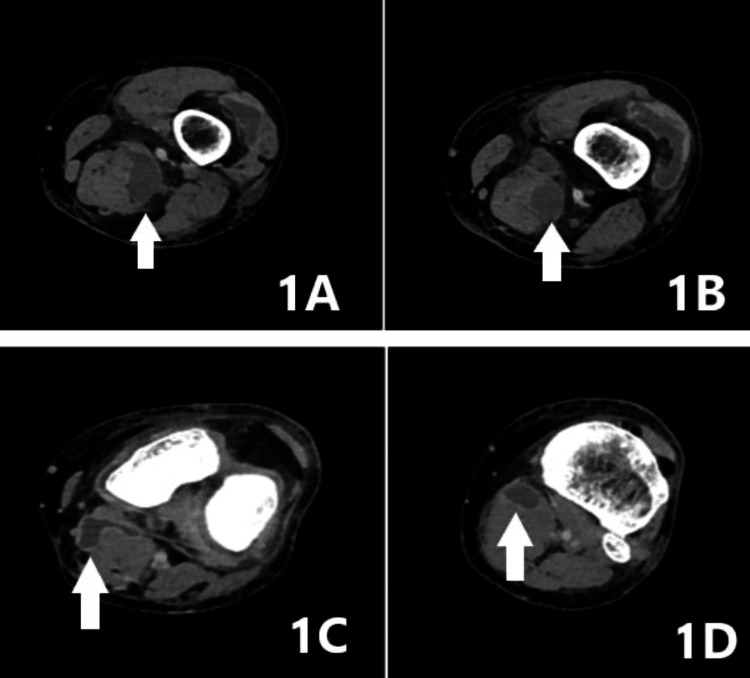
Preoperative CT scan. A. Cyst in the semimembranosus muscle (white arrow). B. Second cyst in the semimembranosus muscle (white arrow). C. Baker’s cyst (white arrow). D. Cyst in the medial head of the proximal gastrocnemius muscle (white arrow).

Plain X-rays of the left knee did not reveal any bony lesions. A CT angiography showed multiple cysts with characteristics indicative of soft tissue abscesses. One was located within the distal third of the semimembranosus (SMM) muscle mass, another anterior to the muscle, and a third within the medial head of the proximal GM muscle. A large Baker's cyst was also identified (Figure 1). An attempt to aspirate the cysts under ultrasound (US) guidance was made on the day of admission, but it was unsuccessful.

The patient underwent urgent surgery approximately 24 hours after admission. No antibiotics were administered prior to the procedure. A posteromedial approach extending from the distal mid-thigh to the proximal third of the GM muscle was performed. The subcutaneous fascia was incised, and the vessels and nerves of the popliteal fossa were meticulously identified and protected. The SMM and GM muscles were dissected, and purulent fluid was drained. The cysts were carefully excised. Thorough debridement and drainage of the surgical field were carried out, and several tissue specimens were collected for cultures and biopsy.

Postoperatively, the patient received intravenous (IV) broad-spectrum antibiotics (meropenem plus vancomycin). Blood cultures and tissue cultures collected during surgery were negative for bacteria. However, biopsy of the cyst tissue from the posterior thigh showed inflammatory infiltration predominantly composed of neutrophil granulocytes. The surgical wound healed uneventfully. During the patient’s hospital stay, infection biomarkers gradually decreased, and he reported mild pain around the knee. Three weeks postoperatively, the patient was discharged afebrile with moderate posterior knee pain. The aforementioned IV antibiotics were continued throughout the three-week hospitalization. No oral antibiotics were prescribed. The laboratory findings on the day of discharge were WBC: 7300, CRP: 1.5, and ESR: 48 (Table 1).

**Figure 2:**
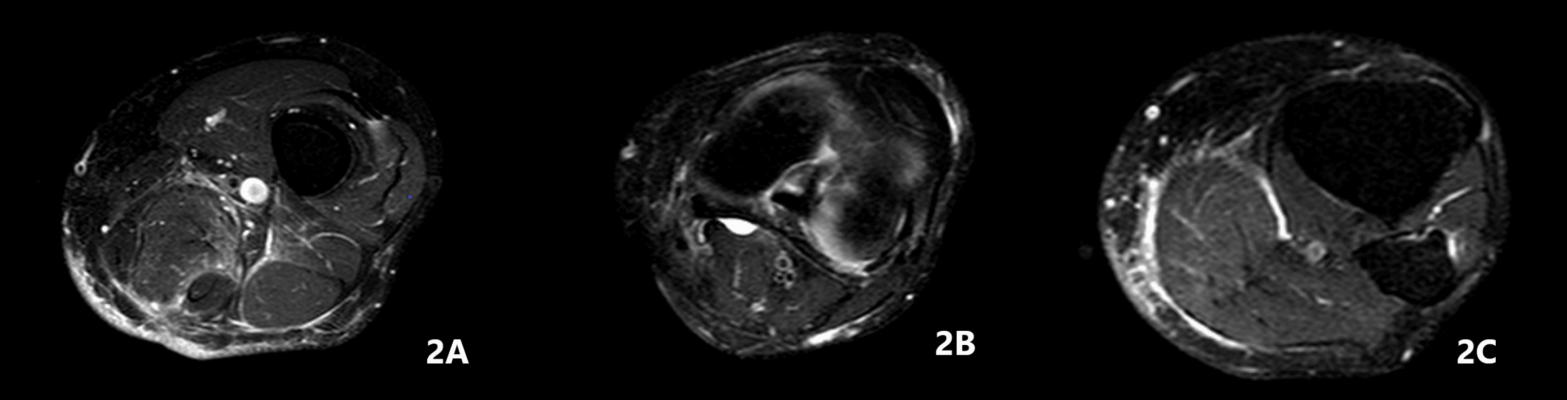
Postoperative MRI scan. A. No cyst observed in the semimembranosus muscle. B. Baker cyst, smaller than in the preoperative CT scan. C. No cyst observed in the proximal gastrocnemius muscle.

Ten days later, the patient returned to the hospital with symptoms similar to those before the surgery. Blood serum tests showed a significant elevation in infection biomarkers. A MRI scan of the left leg with IV gadolinium was performed to evaluate the soft tissue and to exclude any bony involvement not detected in the preoperative CT scan. The examination did not reveal any cyst recurrence (Figure 2).

**Figure 3:**
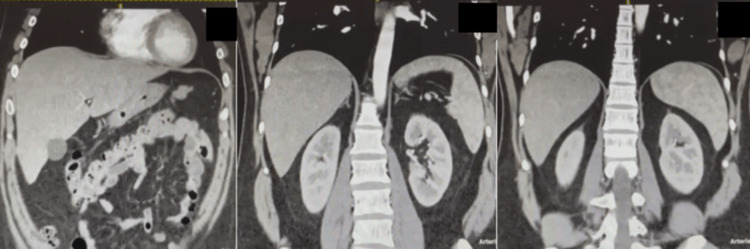
Representative images from the patient’s thoracic and abdominal CT scan. No cystic lesions are evident in the lungs, liver, spleen, or kidneys.

A CT scan of the thorax and abdomen was also performed, and no other septic lesions were found (Figure 3).

Due to the new onset of symptoms and the patient's occupation, specialized tests for rare infectious diseases, such as viruses, Brucella, fungi, and Quantiferon, were conducted. The patient was advised to take oral doxycycline and rifampicin for six weeks. All the aforementioned tests were negative, and the antibiotics were subsequently withdrawn. One month after discharge, the patient experienced two episodes of severe left knee joint effusion with fever. Joint aspiration was performed, and fluid cultures were negative for bacteria.

At this point, a diagnosis of ACS was made. Following the rheumatologist’s instructions, the patient received high-dose corticosteroids, resulting in rapid symptom relief and laboratory improvement. Given the strong association of ACS with IBD and autoimmune disorders, further investigations, including colonoscopy and specific serologic tests, were conducted. Intestinal endoscopy was not consistent with IBD, and only mildly positive antinuclear antibodies (ANA) were identified. Nevertheless, the patient remained symptom-free at the nine-month follow-up and is currently on methotrexate. He remains under rheumatologic surveillance, as the development of an autoimmune disease in the future remains a possibility.

## Discussion

Aseptic cyst syndrome (ACS) was first described by André et al. in 1995 in a case report involving a 25-year-old patient with Crohn’s disease and liver AA [8]. This medical entity consists of deep collections of PNNs and presents with symptoms such as pain, high fever, and leukocytosis. However, cultures taken from the cysts are negative for bacteria, and the condition does not respond to antibiotics. In contrast, CS and immunosuppressant drugs are highly effective [9]. Trefond et al. proposed that the diagnostic criteria for AA include deep abscess(es) visible on imaging, negative blood and tissue cultures, failure to respond to antibiotics, and rapid response to CS [1].

The most common site of involvement is intra-abdominal [1,4,7,10]. Intramuscular locations are much rarer, accounting for approximately 10% of cases [1,9]. The clinical presentation often mimics an infectious disease, including localized pain at the site of the cysts and fever. Our patient exhibited symptoms and signs consistent with infected intramuscular abscesses around the left knee, along with elevated inflammatory markers. However, both blood and cyst cultures were sterile, and biopsy results confirmed inflammation. Furthermore, the patient did not respond to antibiotic treatment but showed rapid clinical improvement following the initiation of CS and methotrexate. These findings align with the typical course of ACS.

The pathophysiology of ACS is not fully understood. Vessels filled with PMNs are sometimes seen at the periphery of AA lesions, though this may simply reflect the influx of neutrophils into the inflammatory site prior to their migration toward the abscess. A defining feature of AA is its prominent neutrophilic component [4]. ACS is often associated with autoimmune conditions such as IBD, RA, hematologic malignancies, and Sweet syndrome [2,5,9,11], though it can also occur in otherwise healthy individuals in about 40% of cases [1,9]. Similarly, in our patient, colonoscopy was negative for IBD, and serological tests did not indicate RA.

ACS often mimics infection, and proper diagnosis is both time- and cost-consuming, frequently leading to unnecessary surgical interventions, with splenectomy being the most common [10,11]. The differential diagnosis includes septic abscesses or soft tissue tumors such as sarcoma. The cornerstone of diagnosis is obtaining appropriate material from the cyst for tissue cultures and biopsy. Negative cultures and a biopsy consistent with an abscess rather than a tumor confirm the diagnosis [1-3,5]. Our patient underwent open drainage of the cysts due to a strong suspicion of infection. However, the cyst content was more solid than expected, which explains the failure of needle aspiration under US guidance. The patient received broad-spectrum IV antibiotics for three weeks. Although treatment led to initial improvement, the clinical status at discharge was not as satisfactory as anticipated. The patient continued to report moderate pain behind the left knee, and while inflammatory markers had decreased, they had not returned to normal. The recurrence of pain and the rise in CRP, WBC, and ESR, along with repeated sterile knee effusions, ultimately pointed to a diagnosis of ACS.

There are no established guidelines for medical treatment. Management typically involves an initial course of CS, followed by immunosuppressive agents such as disease-modifying antirheumatic drugs (DMARDs) or biologics [4,11]. Our patient received CS for three months, followed by long-term methotrexate therapy. This approach proved successful, with no relapse observed at the nine-month follow-up. However, the patient was informed about a potential recurrence rate of 60% [1]. The risk of relapse is higher in cases involving hepatic or skin abscesses and lower in patients with IBD or when colchicine is prescribed [1].

To the best of our knowledge, this is one of the few reported cases of ACS. The absence of underlying IBD or autoimmune disease, along with the rare intramuscular location of the abscesses, makes this case particularly unique. Sharing each case of ACS with the scientific community is essential, given the rarity of this recently identified condition. ACS requires a multidisciplinary approach involving multiple medical specialties to ensure appropriate management.

## Conclusions

ACS is extremely rare and primarily a diagnosis of exclusion. It is commonly associated with autoimmune diseases. When an intramuscular abscess is suspected, aspiration should be attempted first, if possible. If aspiration fails, surgical drainage becomes necessary. Tissue cultures and biopsies are of paramount importance. If cultures are negative and the patient is unresponsive to antibiotics, ACS should be suspected. Prompt improvement with CS and other immunosuppressive medications is typical. Our case highlights the need for a multidisciplinary approach to provide appropriate treatment for this rare condition.
